# Brain abscesses in a patient with a patent foramen ovale: a case report

**DOI:** 10.1186/1752-1947-3-9299

**Published:** 2009-11-25

**Authors:** Fuad Jan, Abdul Moiz Hafiz, Saurabh Gupta, John Meidl, Suhail Allaqaband

**Affiliations:** 1 Department of Medicine, Division of Cardiovascular Disease, University of Wisconsin School of Medicine and Public Health, Milwaukee Clinical Campus, North 12th Street, Milwaukee, Wisconsin, 53233, USA; 2 Department of Medicine, Winthrop-University Hospital, Mineola, New York, USA; 3 Department of Medicine, Division of Infectious Diseases, University of Wisconsin School of Medicine and Public Health, Milwaukee Clinical Campus, North 12th Street, Milwaukee, Wisconsin, 53233, USA

## Abstract

**Introduction:**

Brain abscesses arising from right-to-left cardiac shunting are very rare in adults.

**Case presentation:**

We describe the case of a 47-year-old non-hispanic white male with periodontal disease who developed several brain abscesses caused by *Streptococcus intermedius*. A comprehensive workup revealed a patent foramen ovale with oral flora as the only plausible explanation for the brain abscesses.

**Conclusion:**

Based on this case and the relevant literature, we suggest an association between a silent patent foramen ovale, paradoxical microbial dissemination to the brain, and the development of brain abscesses.

## Introduction

Brain abscesses develop as a result of the contiguous spread of infection from the sinuses, otic or odontogenic primary sources, or as a result of hematogenous spread from distant locations. In 10% to 60% of patients with brain abscess, no underlying source of infection is found. Unlike children with congenital heart disease, minor right-to-left intracardiac shunting due to a patent foramen ovale (PFO) is not recognized as a cause of brain abscess in adults.

## Case presentation

**Figure 1:**
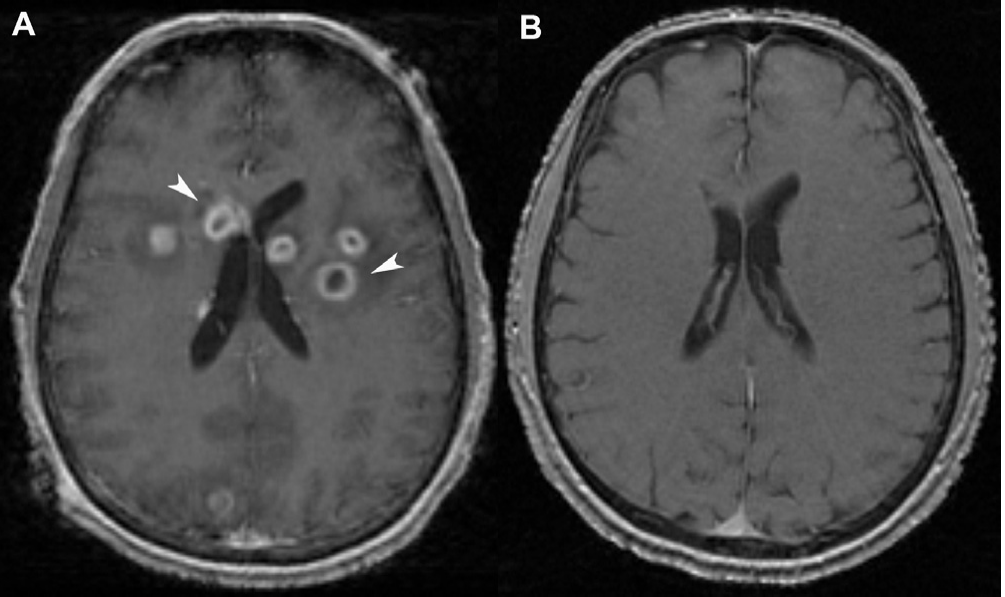
**Magnetic resonance imaging of the brain**. **(A) **T1-weighted images at the patient's presentation, post-contrast showing multiple ring enhancing lesions in the brain (white arrow heads). **(B) **T1-weighted images eight months after treatment showing a resolution of the brain abscesses.

A 47-year-old non-hispanic white male from United States, with a history of alcohol abuse presented to the emergency department of our hospital with nausea, vomiting, fever and severe headache of a two-week duration. On examination, he was found to have an altered sensorium, periodontal disease and low-grade fever. Laboratory evaluation was unremarkable except for a white cell count of 13,400 cells/μl (normal range 4,000-11,000 cells/μl). Magnetic resonance imaging (MRI) of the brain (Figure 1A) showed 15 to 20 ring enhancing lesions in both the cerebral hemispheres and the midbrain.

**Figure 2:**
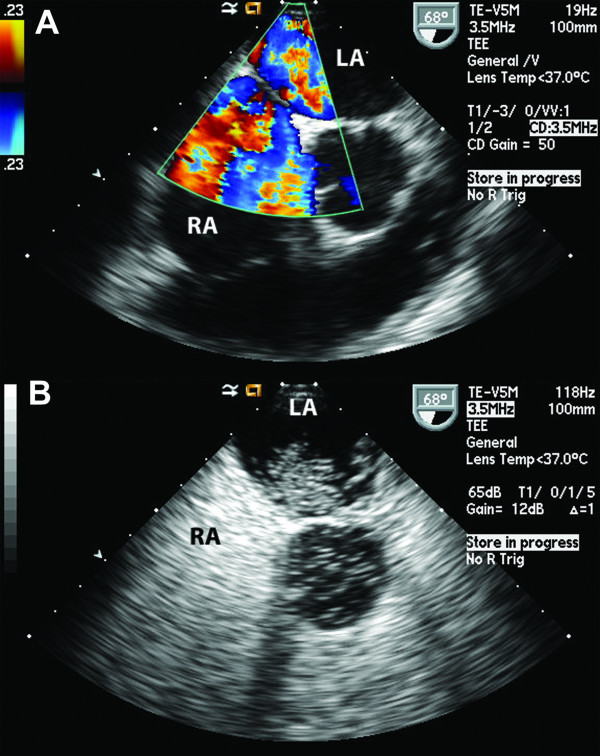
**Transesophageal echocardiography during the patient's initial hospitalization**. **(A) **A bidirectional interatrial shunt seen through the patent foramen ovale. RA, right atrium; LA, left atrium. **(B) **Injection of agitated saline through an upper extremity peripheral vein showing the appearance of bubbles crossing from the right to the left atrium. RA, right atrium; LA, left atrium.

Craniotomy and biopsy revealed multiple abscesses positive for *Streptococcus intermedius*. A search for the primary source of infection led to an extensive workup that included tests for neurocysticercosis, human immunodeficiency virus, tuberculosis, rheumatology panel, and computed tomography of the chest, abdomen and pelvis, all of which returned normal results. A transesophageal echocardiogram to look for vegetations (Figure 2A), revealed a moderate-sized PFO with a bidirectional shunt, which was confirmed with the use of agitated saline bubble contrast echocardiography (Figure 2B).

Our patient's PFO was believed to be the pathway for his cerebral abscesses. He was started on a prolonged course of antibiotics and discharged from the hospital after two weeks. The follow-up MRI scans improved gradually (Figure 1B) and he experienced a complete clinical recovery. Eight months later he underwent a closure of the PFO with a GORE HELEX device.

## Discussion

PFO has a prevalence of 25% in the general population [1], and it has been proposed that oropharyngeal bacteria may access the brain vascular system through a PFO [2]. Although congenital intracardiac shunts are known to be a path for pediatric brain abscesses, and extracardiac shunts such as pulmonary arteriovenous malformation are recognized to be pathways in adults, clinically silent intracardiac shunting due to PFO is not a recognized source of septic brain emboli in adults. The spectrum of organisms found in brain abscesses reflects the range of underlying primary sources of infection. Although in 10% to 60% of patients with brain abscess no underlying source of infection is found, a PFO may precipitate the development of brain abscess in these cases [3].

Our patient's brain abscesses had no precipitating factors other than a PFO, which was detected by an agitated saline bubble contrast echocardiography. The association of the brain abscess with dental sepsis is suggested by the isolation of oral *Streptococcus milleri *group of organisms (*Streptococcus intermedius *in our case). Isolated cases in the literature have suggested that all patients in whom an oropharyngeal pathogen is isolated from brain abscesses must be screened for a PFO by transesophageal echocardiogram [4]. Management options include medical therapy with antiplatelet agents or anticoagulation (for stroke prevention) and surgical or percutaneous closure of the defect.

## Conclusion

A minor PFO is not recognized as a source of brain abscesses in adult patients. However, it may become a source of brain abscesses in rare cases. This case highlights a possible link between a silent PFO and the development of brain abscesses, particularly with oral flora. To the best of our knowledge, there were very few instances in the past where such a possibility was considered. As such, this possibility should be kept in mind when evaluating patients with brain abscesses in whom the underlying cause seems unclear.

## Consent

Written informed consent could not be obtained because the patient was lost to follow-up. Every effort has been made to conceal the identity of the patient and we believe that the patient and their family would not object to publication of this case report.

## Competing interests

The authors declare that they have no competing interests.

## Authors' contributions

FJ, JM and SA cared for the patient while he was admitted in the hospital. They later reviewed, edited and approved the final draft of the manuscript. AMH and SG reviewed the charts and the literature, and also drafted the manuscript.
